# Ophthalmology

**Published:** 1895-09

**Authors:** Alvin A. Hubbell

**Affiliations:** Professor of ophthalmology and otology in the Medical Department of Niagara University, etc.


### Progress in Medical Science.

### OPHTHALMOLOGY.

By ALVIN A. HUBBELL, M. D.,
Professor of ophthalmology and otology in the Medical Department of Niagara University, etc.

### TREATMENT OF DETACHMENT OF THE RETINA.

DR. TERSON, of Toulouse, is of the opinion that electrolysis, at present, appears to be the best method of treating detachment of the retina. Some practitioners are in the habit of adapting to this affection the measures resorted to for the cure of hydrocele, introducing simultaneously into the eye two needles connected with the opposite poles of an uninterrupted current battery (bipolar electrolysis, Schoeler). Others, as in the treatment of aneurisms and nævi, introduce into the eye a single needle, connected with the positive pole (positive electrolysis, Abadie, Gillet de Grandmont). Others, again, introduce a needle connected with the negative pole (negative electrolysis, Van Moll).

The positive pole is the one that should be introduced into the eye, in order to let the wound close as rapidly as possible, so as to prevent the vitreus from escaping, on the same principle as positive electrolysis is to be preferred in the cure of aneurisms, in order to obviate secondary hemorrhage. In addition, electrodes must be used which are not attacked by the products of electrolysis, and which, on the other hand, are sufficiently rigid to penetrate the sclerotic of a hypnotic eye presenting detachment of the retina. The only substance which fulfils these conditions, being at the same time remarkably easy to asepticise, is iridised platinum. The only drawback is that this alloy does not take the bright polish which facilitates penetration into the eye.

The author uses positive electrolysis, the capital point in which is to measure the strength of the current by means of a very sensitive galvanometer without oscillation. He has succeeded by this means in keeping up a constant current of five milliamperes during the whole time the application lasted, which did not exceed one minute.

Since July, 1894, the author has used this method seventeen times in operating for the relief of detachment of the retina among nearly 1,700 patients under his observation. Of these, five have been operated upon so recently that it is impossible to form an estimate of the result. In the other twelve cases of detachment, all of which were of a very serious nature, there was aggravation in one, dating from nearly three years, no effect in two, ephemeral improvement in three, improvement persisting in a very promising manner in five, and, lastly, in one, immediate recovery, which has now been maintained during nine months, and which must be attributed solely to the intervention, seeing that this was the only treatment that had ever been applied.

The patient in the last case was a woman, thirty-eight years of age, who for a week had presented extensive myopic detachment of the retina of the right eye, involving rather more than one-third of the membrane in the infero-external region, associated with general disturbances in the vitreus, abnormal depth of the anterior chamber and considerable weakening of the pupillary reflex. Only a very slight perception of light remained in the non-detached portions of the retina; there was well-marked hypotonus. Forty-eight hours after the application of positive electrolysis, with a current of 5½ milliamperes during one minute, the vitrous resumed its transparency and the retina was reattached everywhere, leaving old spots of disseminated choroidal atrophy visible, with lesions of the macula. Vision, which before was almost *nil*, had increased to $\frac{1}{10}$ with a correcting lens of —12D. The visual field had become normal. This result has been fully maintained for nine months. These facts seem to justify the following conclusions:

1. Positive electrolysis should be resorted to in recent cases of detachment of the retina, and is the more likely to be successful the sooner after the initial symptoms it is undertaken.

2. This method of treatment does not in the least interfere with recourse to various medical measures, suggested by lesions of diathetic origin, which a long experience has proved to possess considerable palliative value.—*The Medical Week* (Paris), May 17, 1895.

---

### OPERATION FOR SECONDARY CATARACT.

Dr. Wicherkiewicz, of Posen, holds that there are two varieties of secondary cataract—namely, a membranous form, complicated by iridal synechia ; the simple form, consisting merely in the thickening of the capsule of the lens without iridal adhesions. In the first case, when the pupil is not entirely obstructed, attempts should first be made to extract the entire membrane, with the help of a pair of forceps, through a small peripheral incision in the cornea. If this maneuver does not immediately succeed, care must be taken not to pull too hard, recourse being had in such cases, as when the pupil is completely obstructed, to iridotomy, a medium triangular incision being made into the iris with a pair of de Wecker's scissors. In the second category, that is to say, in simple secondary cataract, discission is sufficient ; but, in order to prevent all chance of infection, the puncture should be made, not into the transparent cornea, but into the peripheral portion of the cornea, in the scleral limbus, at a distance of one millimeter from the iris, a Knapp's knife being used for the purpose. The author proposes to describe this operation as scleronyxis anterior.—*The Medical Week* (Paris), May 17, 1895.

---

### ON THE RELATION OF RETINITIS ALBUMINURICA TO THE INDUCTION OF PREMATURE LABOR.

Mr. Simeon Snell, of Sheffield, has read a short paper before the North of England Obstetrical and Gynecological Society, in which he defended the practice of inducing premature labor in certain cases of albuminuric retinitis. He says :

The seriousness of the renal complication is increased by the sight-failure, which is present in a certain number of cases. The association of retinitis with Bright's disease is indicative of a very limited period of life, frequently only months. With the albuminuria of pregnancy the retinitis is of less grave import in this respect, but, as far as vision is concerned, it is attended with very serious results. Dr. Culbertson has collected the cases of albuminuric retinitis which have been recorded, and finds that 23.33 per cent. have terminated in blindness, 58.25 have resulted in only partial recovery of sight, and but 18.54 have recovered sight. Silex, who has followed twenty-six patients for a long period, gave the following results : Eleven recovered vision above one-sixth, ten below this, and five were nearly blind, this being due to optic atrophy, choroido-retinitis and detached retina, which showed itself late in two cases.

The retinal affection may show itself at almost any period of pregnancy, and infrequently during the first three months. The appearances in the fundus are generally well pronounced. The margins of the optic discs may be hazy and ill-defined ; numerous white patches in the macular region, with some hemorrhages, may all be observed. In other cases blindness or great impairment of sight may be present, with an absence of ophthalmoscopic signs. In a case recently, which Mr. Richard Favell kindly allowed me to examine, and in which labor was induced for eclampsia at term or nearly so, the young woman was apparently quite blind, but yet there was an absence of ophthalmoscopic signs. On the other hand, the gravity of the retinal affection, as shown by the changes in the fundus, is not always commensurate with the defective vision complained of by the patient, and for this reason it is desirable that pregnant women, with albumen in their urine, should at intervals undergo ophthalmoscopic examination. The gravity of the complication has been sufficiently dealt with.

It has frequently been found that when labor terminates, improvement in sight is apparent, and in this way the induction of abortion came to be advocated. Excellent results have followed its adoption. It has been called for occasionally in the author's own practice.

Mr. Snell concludes as follows :

1. For retinitis, appearing before or about the sixth month, induction of labor should be recommended.

2. That when it shows itself only in the last few weeks it may often be unnecessary, but that each case must be judged by the severity of the affection.

3. That a case in which retinitis has shown itself in one pregnancy should be carefully watched both as to the presence of albumin in the urine and as to the eye-affection, and treatment adopted accordingly.—*British Medical Journal*, June 22, 1895.

---

### THE PARALLAX TEST FOR HETEROPHORIA.

Dr. D. A. Duane, of New York, says, that having used this test for eight years, and upon a great number of cases, he is thoroughly convinced of its utility and desires to recommend it to those who busy themselves with the consideration of anomalies of the ocular muscles.

To conduct this test he places the patient in the primary position, with head erect and eyes directed straight forward or slightly below the horizontal plane (this latter especially in making the test for near points). The object of fixation, which should be twenty feet distant, may be a candle flame, but preferably is a white spot 1 or 2 cm. in diameter, upon a dull black surface of some considerable extent. By this arrangement all danger of projecting the image upon a surface beyond is done away with, and the chance of confusion with surrounding objects is prevented. The patient's gaze being directed fixedly at the spot, a card is placed before one eye and passed alternately from that to the other—the patient being at the same time asked whether the spot appears to move, and, if so, in what direction. If it remains perfectly stationary there can have been no deviation behind the card, and the position of fixation of both eyes is perfect. If, however, the spot moves, it must occupy a different position as seen by the two eyes, *i. e.*, there is really a diplopia present which our method of observation has unmasked. Thus, if, on uncovering the left eye, the object (which was previously seen by the right eye and is now seen by the left) appears to move to the patient's left, there is really a homonymous diplopia (homonymous parallax), which differs from ordinary diplopia only in the fact that the two images are seen alternately instead of at the same time ; if the object seems to move to the right, there is crossed diplopia (crossed parallax); if the object moves down, the eye must have been higher behind the screen (left hyperphoria, left parallax); and if the object moves up, the left eye must have been lower behind the screen (right hyperphoria, right parallax). In order to determine the amount of this alternate diplopia, he places prisms of the appropriate direction and strength before one eye until the movement is abolished. Thus, supposing that when the left eye was uncovered, the object seemed to move down and to the left, (homonymous and left parallax, indicating a condition of hyperesophoria,) two prisms are placed before this eye, with their bases respectively down and out, and increased in strength until the movement has become *nil.* The strength of the prism having its base down will measure the degree of hyperphoria, and that of the prism having its base out will indicate the degree of esophoria present.

For near points the test is made in the same way, a small dot on a rather large card being employed, and the movement of the dot upon the card (and not of the card itself projected against some distant object) being observed.

Of any test thus put forward it must be asked how far it fulfils the requirements of applicability, precision, accuracy and utility. As regards the first requirement, he says that in his hands the test has proved to be almost universally applicable.

As regards precision, the test leaves nothing to be desired. A prism of $\frac{1}{2}°$ suffices to produce or to neutralize quite a decided lateral parallax; and a vertical movement, corrected by a prism of even $\frac{1}{8}°$, is clearly noticeable.

As regards accuracy, he has become convinced from the constancy of the results which the method affords, that it is one of the most reliable of all that we have at our command. And this, in fact, constitutes its reason for existence.—*Archives of Ophthalmology*, Vol. XXIV., No. 2, 1895.

---

### MASSAGE OF THE EYE.

Dr. Parenteau, of Paris, holds that massage of the eye and its appendages constitute a method of treatment which is worthy of being tried and applied in a large number of affections.

Maneuvers forcible enough to determine traumatism may be beneficial in lesions of the eyelids, such as cysts, chalazion, engorgements, and the like.

In ocular affections, properly so-called, he regards them as too dangerous.

Massage with medicinal substances possesses the double advantage of assisting the penetration into the eye of the ointments or powders introduced under or applied to the surface of the lids, and of bringing about a quicker subsidence of the congestion of the eye and its envelopes. It is especially indicated in phlyctenular kerato-conjunctivitis, corneal leucomata, iritis, episcleritis and irido-choroiditis.

Simple massage, executed with the aid of the fingers or special instruments, without the addition of drugs, is called for whenever there is a muscular lesion of peripheral origin, or vascular disturbances. Massage is contra-indicated in progressive myopia of high degree, considerable hypotonus and incipient opacity of the crystalline lens.

The *modus operandi* varies from slight touch to strong pressure, and includes also percussion of the tendons, in affections involving the muscles of the eye. The motion should be radial and circular in corneal or iridal lesions, longitudinal in muscular lesions and vertical in episcleritis and irido-choroiditis.—*The Medical Week*, May 24, 1895.

